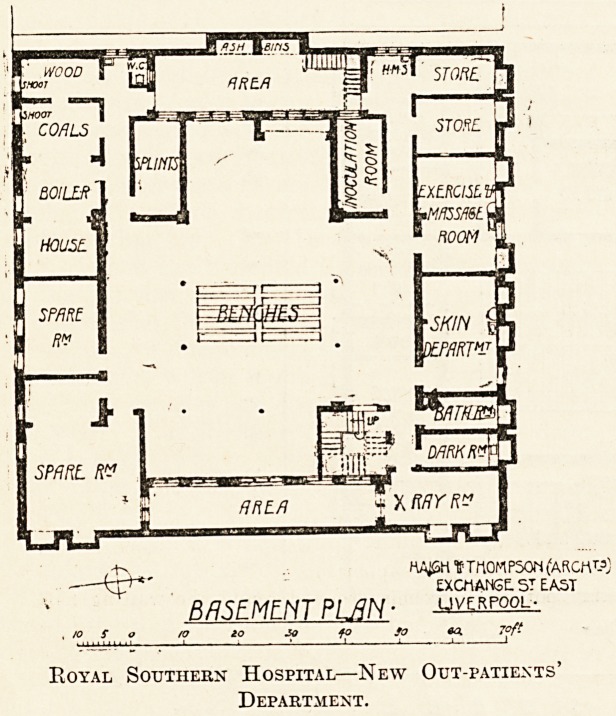# Royal Southern Hospital, Liverpool

**Published:** 1910-12-31

**Authors:** Allen Naldrett

**Affiliations:** Superintendent and Secretary, Royal Southern Hospital, Liverpool.


### SPECIAL INSTITUTIONAL ARTICLES.

### ROYAL SOUTHERN HOSPITAL, LIVERPOOL.

### NEW OUT-PATIENT DEPARTMENT.

By Allen Naldrett, Superintendent and Secretary, Royal Southern Hospital, Liverpool.

FOR many years past, in consequence of lack of funds, the committee of the Royal Southern Hospital has regretfully been compelled to admit that its out-patient department has been an object-lesson of much that ought not to be. Nor is it to be wondered at that this department, opened with the hospital in the year 1872, has outgrown the ideas of its promoters, when it is known how enormously the population has increased, and that the district which it serves now numbers some 340,000 persons, indicated by an increase in the attendances from 12,500 in 1873 to 48,000 last year. Constructionally, also, it reflects no discredit upon its designers; yet viewed in the light of forty years' advance in science and hospital construction—the intervening period of asepsis, the knowledge of the dangers of dust, improved sanitation and ventilation—it cannot now but be considered as hopelessly out of date and inefficient.

#### THE OLD BUILDING.

A brief description of the department as it has existed hitherto, for comparison with that of the new building, may serve to illustrate these advances. It consists of three rooms—a waiting hall, surgical dressing-room, and a consulting-room.

The waiting hall is a windowless room (44 ft. by 25 ft.) lighted only by glass panels in a boarded roof supported by exposed wooden rafters. For ventilation it relies for its inlet upon the entrance doors, the lavatories (which communicate directly with the hall), and some small "Tobin" tubes—the outlet is by an extractor in the centre of the roof. It is floored with pitch pine with open joints.

The surgical dressing-room (28 ft. by 18 ft.) has large sash windows on one side only, is fitted with germ-harbouring fitments of wooden drawers and cupboards, with similar flooring to the waiting hall.

The consulting-room (13 ft. by 13 ft.) receives light and ventilation from the roof only and is floored with wood blocks.

The overcrowded and unhealthy conditions existing in the small surgical dressing-room, with five resident medical officers, two nurses, and two or three students all dressing patients at one time, can readily be imagined.

It will be seen also how hampered has been the work of the honorary medical officer with but one badly lighted, and worse ventilated, consulting-room. On a busy day it has been quite impossible for an honorary and his assistants to get through the work without making their examinations in the open waiting-hall. Crowded into this small hall might be seen from a hundred to a hundred and fifty persons—males, females, and children—who, whilst waiting their turn for treatment, have had to witness the examination and dressing of their fellow sufferers. The least imaginative person will not fail to realise how necessary had become, in the interests of patients and doctors alike, the provision of increased and modern accommodation.

The rapid increase of population, the growth of the docks, the quicker shipping traffic due to the introduction of machinery for loading and unloading—all tending to a larger influx of casualties—made it evident long since that this out-patient department must ultimately be extended. With this object in view, the President (Mr. William Adamson) nearly twenty years ago collected a sum of money for the purchase of properties opposite the hospital to furnish a site.

### The Appeal for Funds.

The site provided, private appeals were made for donations to commence the building, but, in consequence of the many calls upon the hospital's friends and the

Royal Southern Hospital—New Out-patients' Department.

charitable public of Liverpool, these met with but poor response. It was not until 1907, when the Liverpool Corporation, in compliance with an application from Mr. John O. Strafford, the Honorary Treasurer, realising the pressing necessity for the extension, obtained Parliamentary powers to grant a sum of £4,000, that the scheme approached realisation.

This welcome donation gave to the movement that impetus which enabled Mr. Strafford to announce within four months that he had collected the sum of £4,500, the additional amount required to cover the cost of the building.

Mr. Alfred Culshaw, the hospital's architect, was appointed assessor, and a competition among Liverpool architects resulted in his award being given to a design by Messrs. Haigh and Thompson. The first brick of the new building was laid on September 20, 1909, and the builders, Messrs. Jones and Sons, of Liverpool, are to be congratulated on the excellence and expedition of the work which was satisfactorily completed within twelve months.

### The "William Adamson" Out-patient Department.

Although too often there is not much in a name or the individual bearing it, this is not so with the veteran hospital worker, Mr. William Adamson, the president of the hospital, to whom this building is dedicated. For nearly fifty years he has laboured with indomitable zeal and energy, ever giving the hospital his first consideration and its patients his earnest care. Through rough and smooth times he has guided its councils and how much also he has had its financial interests at heart can be abundantly testified by many who, knowing and feeling his devotion, have been unable to withstand his solicitations. His acts of kindness, and help extended to poor patients upon his visits to their bedsides, have endeared him in the hearts of many hundreds, and by none more than they will be shared the feelings of love and esteem which prompted the committee to give the building the name of "William Adamson" to stand as a lasting memorial of his good work and worthy example.

### Details of the Building.

The new building is quite separate from the hospital proper, and, although substantially built, of good appearance, and containing all necessary accommodation, it has the further advantage of being economical in cost. It is built of brick, the outside being of red Ruabon with Portland stone dressings, the two central piers in the front elevation being surmounted with stone finials which Mr. Adamson purchased from the Liverpool Exchange Company and presented for the purpose. These finials originally occupied a position at the summit of the Exchange buildings and were removed some years ago as they were considered to be a source of danger. In their less lofty, but more imposing, position they will serve as remembrancers of their donor in his dual capacity as president of the hospital and chairman of the Liverpool Exchange Company.

The large central waiting hall, planned to accommodate 200 persons, is approached by two entrances—one for patients the other for the staff. The passage way from the patients' entrance, past the space allotted for the porter and inquiry officer, is an inclined plane and admits of easy negotiation by lame patients and ambulance carriages. The main walls of the hall are carried up considerably higher than the surrounding rooms, with large clerestory windows on all sides, providing a maximum of light and natural ventilation. Another feature of the hall is the semicircular plastered ceiling free from any projections for harbouring dust, which hygienic principle has been observed throughout—the mouldings of all woodwork and the angles in all the rooms being rounded.

On the south side are situated an isolation-room, with a separate exit to the street, a surgical dressing-room, three consulting-rooms, and a room containing foot and arm baths. This accommodation is repeated on the north side with the substitution of a dental-room for the isolation-room. Retiring-rooms and lavatories for doctors and nurses and the dispensary are placed on the east side. On the west there is the operating theatre, with recovery-room adjoining, a dark-room for eye, throat, and nose examinations, and a consulting-room for these special departments.

The series of foot and arm baths above referred to are of recent introduction into hospital plans and have proved most useful for the removal of old dressings, saving the time of nurses and dressers and relieving the dressing-rooms during this slow and painful process.

The central hall and all the rooms where surgery will be practised are floored with Terrazzo, extended up the walls to form a dado, and the consulting-rooms with maple, in narrow widths, tongued and grooved, and nailed directly on to concrete, forming almost jointless and solid floors.

In a well-lighted and airy basement there is another waiting hall, around which are situated the *x*-ray room, developing-room, skin department, consulting and bath rooms, massage and exercise room, plaster-bandage room, inoculation department, splint room, crye-therapy or freezing-room, the heating chamber and fuel stores.

The inoculation department is the first to be introduced into any provincial hospital and from its inception may be anticipated an incalculable boon to the city. Not only will the wards through its agency be relieved of cases which now occupy beds for unnecessarily long periods, during the healing processes of sinuses, ulcers, and the like, but persons predisposed to any of the diseases for which vaccines are now known to give immunity—as surely as the more commonly known vaccine gives immunity against smallpox—will be able to guard themselves against the attacks of tuberculosis and other incipient diseases. Furthermore, it is proposed that these vaccines, prepared in the hospital laboratory by Dr. Moore Alexander, the bacteriologist, under whose charge this department will be conducted, shall be placed at the disposal of the medical profession for the inoculation and treatment of their patients. By this means the department, besides being a boon to all classes, will also be self-supporting.

One other innovation is worth mention. The roof of the rooms around the waiting-hall is flat and paved with Trinidad asphalt, forming an excellent roof garden, approached by a spiral iron staircase. The hospital being near the docks, a long distance from any open space, and the off-duty time on some days but limited, this roof garden will satisfy a long-felt want by providing a place in the summer time for the nurses to spend these leisure moments in the open air.

### A CRITICAL DESCRIPTION OF THE NEW OUT-PATIENT DEPARTMENT.

This new addition to the Royal Southern Hospital occupies a site quite apart from the hospital proper and bounded on three sides by streets. A more appropriate site could hardly, we think, have been found for the purpose, and an obvious advantage of such a site is that the entrance for the patients could be arranged in one street with the exit in another, thus attaining what is one of the most important things in planning an out-patient department—that is, the automatic guiding of the patients from entrance to exit without unnecessary crossing. In this case, however, this advantage has apparently been lost sight of, and there is one comparatively narrow doorway and passage which serves for both entrance and exit.

The scheme of the work it is not quite easy to follow. On one side of the large entrance hall there are three consulting-rooms for male patients, all adjoining and communicating with each other, an examination-room, and a large male dressing-room. On the other side are three large consulting-rooms for female patients, likewise *en suite*, and a large female dressing-room. With the exception of the examination-room for the male patients, there are no examination-rooms at all attached to any of the consulting-rooms, and two of these consulting-rooms on each side are approached from a narrow passage leading to the baths and lavatories. On the west side there is an operation-room, a recovery-room, a consulting-room for the special departments, and a dark-room for eye, throat, and nose examination. Apparently the eye-room will have to suffice for all the work of this department. On each side there is a set of foot and arm baths; as these are intended, we presume, for surgical patients, we should have thought that they would have been better placed in the dressing-rooms. As it is, apparently a patient has to go to the further end of the building to have old dressings removed, and then has to go back to the dressing-room to have a new dressing put on. The sanitary offices for patients communicate directly with the corridor, and there is no attempt to disconnect them from the general air of the department.

In the basement there is a large waiting-hall, lighted at each end from open areas, in which are to be found the electrical and skin department, exercise and massage room, baths, stores, boiler-house, inoculation-room, and splint store. The inoculation department is the first to be introduced in any provincial hospital, and it is hoped that the department, besides being useful to the hospital, will be to some extent self-supporting.

The roof of the rooms grouped round the waiting hall is flat and has been laid with asphalt, and is intended to serve as a roof-garden for the nurses.

The plans are the work of Messrs. Haigh and Thompson, of Liverpool.

## Figures and Tables

**Figure f1:**
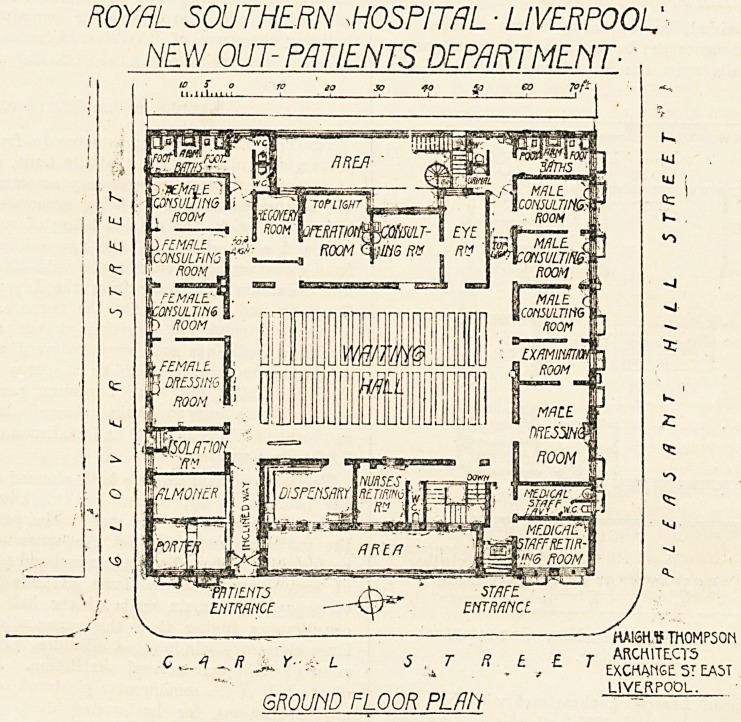


**Figure f2:**